# Supplementary material for: ACE2 expression in adipose tissue is associated with COVID-19 cardio-metabolic risk factors and cell type composition
Source: medRxiv. 2020 Aug 14:2020.08.11.20171108. Preprint. [Version 1] doi: 10.1101/2020.08.11.20171108 (PMC7430606; doi:10.1101/2020.08.11.20171108)
Supplement: Supplement 2020 [file 93049-2020.08.11.20171108-1.pdf]

SUPPLEMENTARY INFORMATION

ACE2 expression in adipose tissue is associated with COVID-19 cardio-metabolic risk factors and cell type composition

Julia S. El-Sayed Moustafa[1*], Anne U. Jackson[2*], Sarah M. Brotman[3*], Li Guan[4*], Sergio Villicaña[1], Amy L. Roberts[1], Antonino Zito[1,5,6], Lori Bonnycastle[7], Michael R. Erdos[7], Narisu Narisu[7], Heather M. Stringham[2], Ryan Welch[2], Tingfen Yan[7], Timo Lakka[8,9,10], Stephen Parker[4], Jaakko Tuomilehto[11,12,13], Francis S. Collins[7], Päivi Pajukanta[14], Michael Boehnke[2], Heikki A. Koistinen[15,16,17], Markku Laakso[18,19], Mario Falchi[1], Jordana T. Bell[1], Laura J. Scott[2**], Karen L. Mohlke[3**] and Kerrin S. Small[1**]

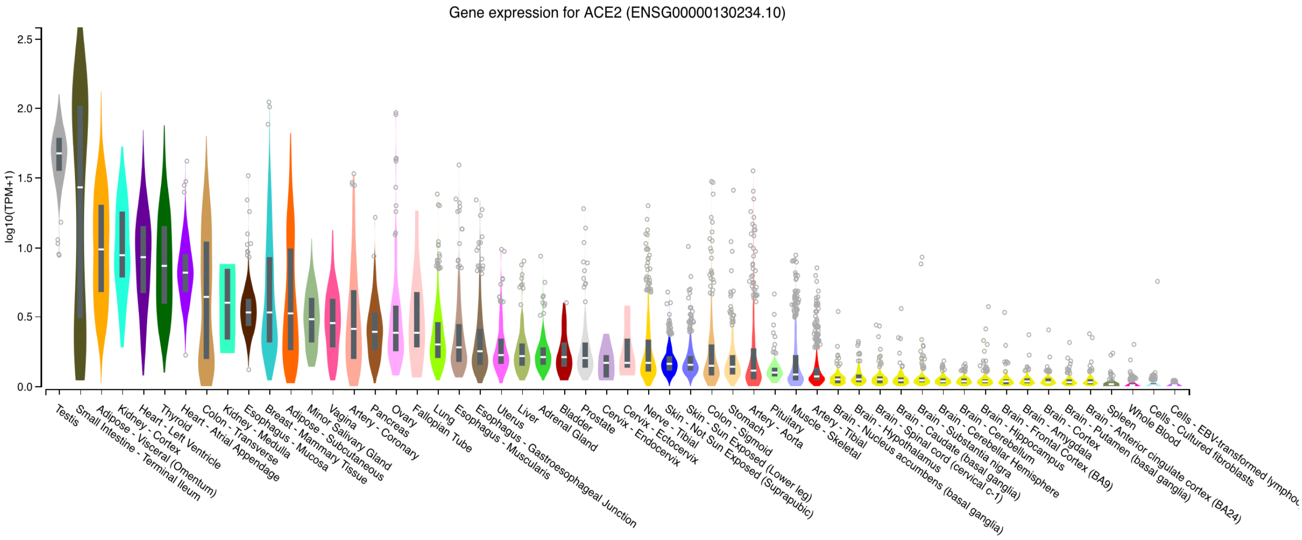

Supplementary Figure 1: Gene expression levels of *ACE2* across 54 tissues in the GTEx resource. Expression levels plotted as log10(TPM+1).

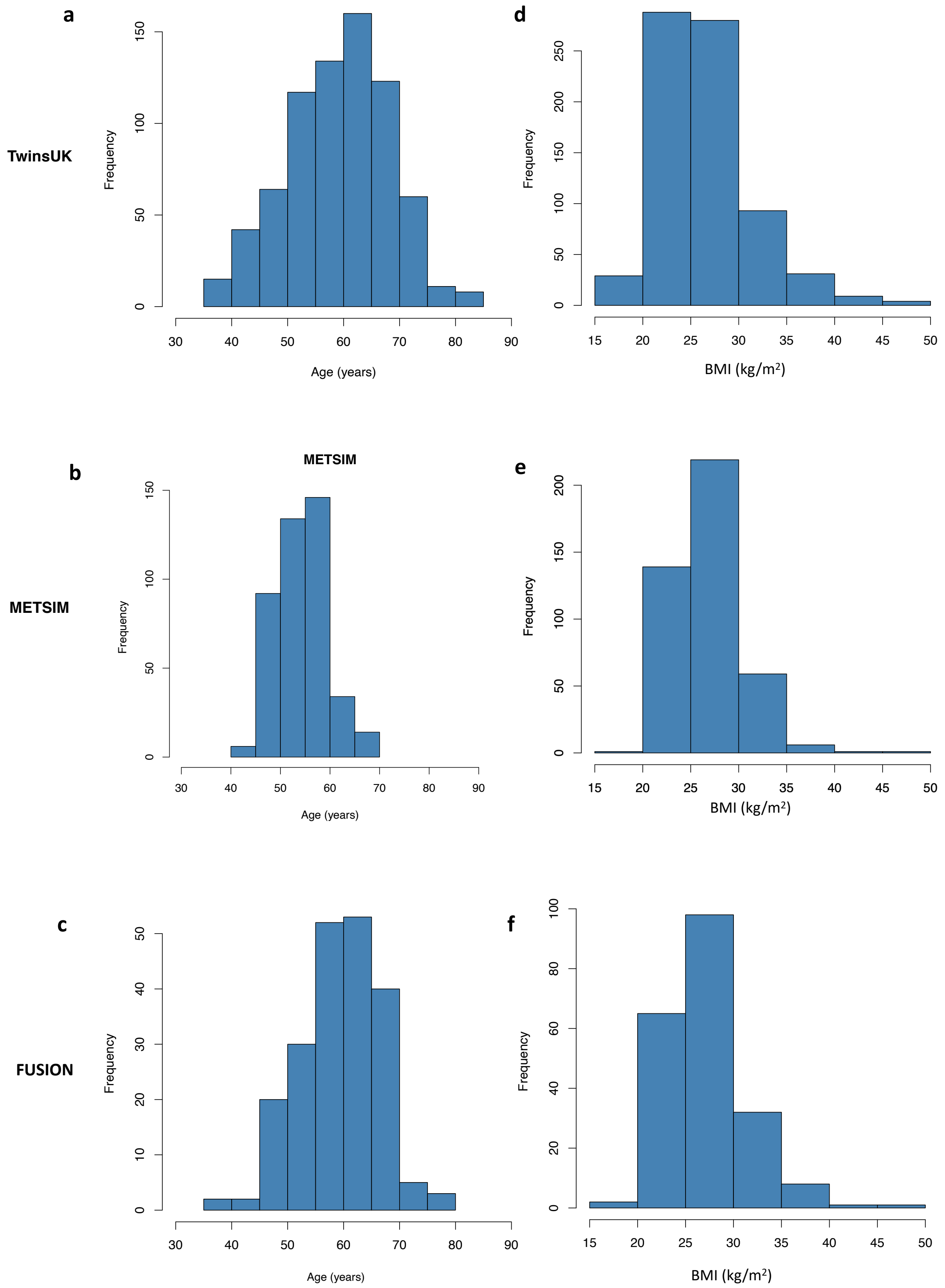

Supplementary Figure 2: Age and BMI distributions in the TwinsUK, METSIM and FUSION studies. a-c) Age distribution in TwinsUK, METSIM and FUSION studies. d-f) BMI distribution in TwinsUK, METSIM and FUSION studies. Plots include only non-diabetic subjects.

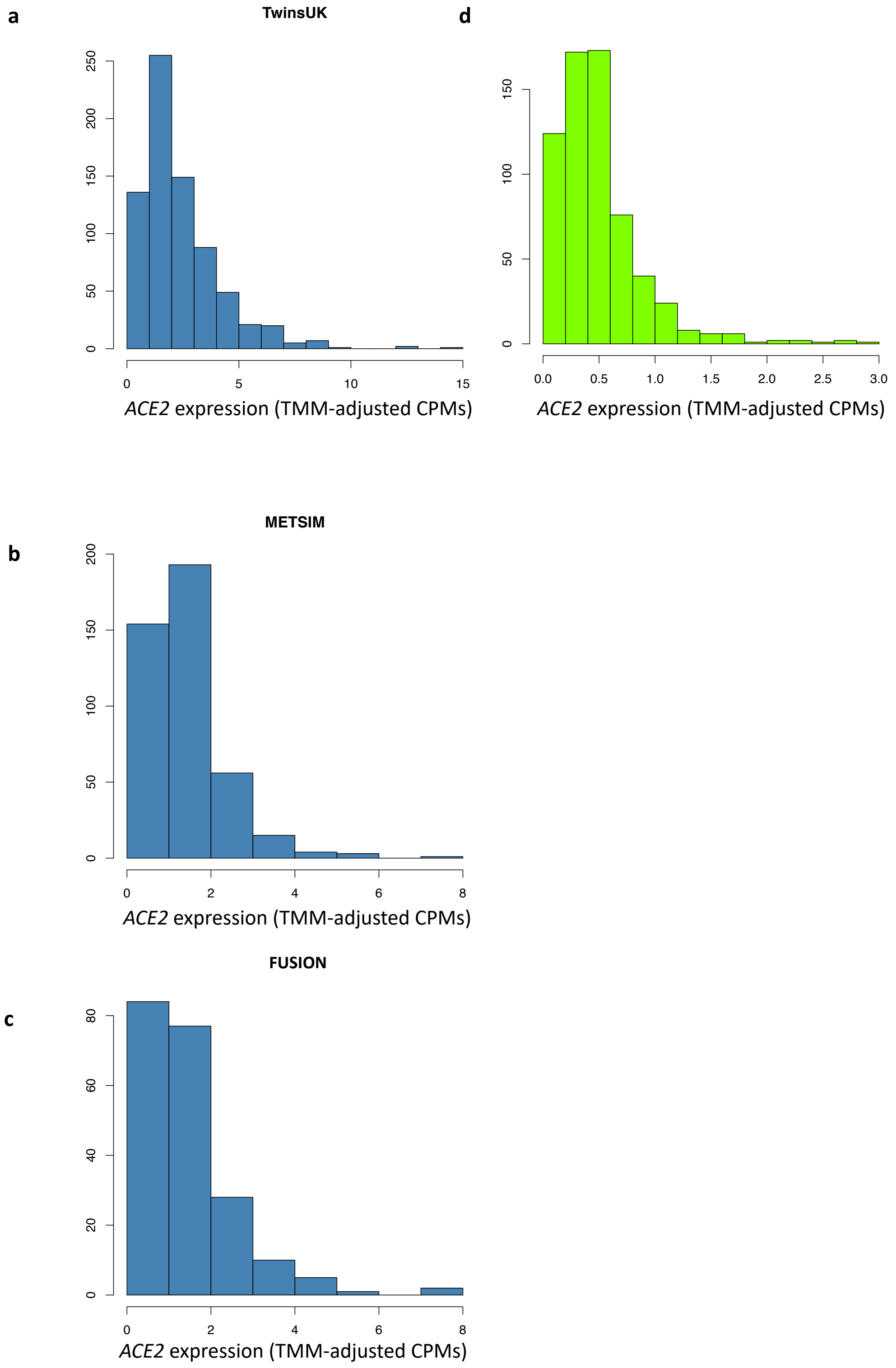

Supplementary Figure 3: *ACE2* expression levels across studies and tissues. *ACE2* expression is plotted as TMM-adjusted counts per million. a) TwinsUK adipose tissue b) METSIM study adipose tissue c) FUSION study adipose tissue d) TwinsUK skin.

Expression of *ACE* and *TMPRSS2* across TwinsUK tissues

We also assessed expression of *ACE2* in skin, lymphoblastoid cell lines (LCLs) and whole blood in TwinsUK. In matched samples from the same subjects in TwinsUK, *ACE2* expression was lower in skin than in adipose tissue (Supplementary Figure 3), and undetectable in lymphoblastoid cell lines and whole blood.

Beyond *ACE2*, two other components of the RAS system of interest are angiotensin-converting enzyme (*ACE*), and transmembrane protease, serine 2 (*TMPRSS2*). ACE plays a central role in regulation of blood pressure[25], while TMPRSS2 has received attention since its identification as a cofactor aiding viral invasion by SARS-CoV-2[7]. We therefore explored their expression and correlation patterns in the TwinsUK multi-tissue sample. *ACE* expression was detected in all four tissues, while *TMPRSS2* expression passed our filters only in skin. When comparing normalised, non-transformed gene counts per million (TMM-adjusted CPMs; TMMAdjCPMs), *ACE* had higher expression levels than *ACE2*, with expression much lower in LCLs and whole blood compared to adipose tissue and skin (median[SD] *ACE* adipose = 41.65[14.02]; median[SD] *ACE* skin = 33.30[16.50]; median[SD] *ACE* LCL = 0.17[0.23]; median[SD] *ACE* whole blood = 3.53[1.70]) (Supplementary Figure 4). *TMPRSS2* expression in skin was also higher than that of *ACE2* (median [SD] *TMPRSS2* skin = 18.63[16.42]). We observed an inverse correlation between *ACE2* and *ACE* expression in adipose tissue (Spearman Rho = -0.19; $P = 8.40 \times 10^{-5}$) but not skin (Spearman Rho = -0.03; $P = 0.53$). No correlation was observed between *ACE2* expression in adipose tissue and skin in the same subjects (Spearman Rho = -0.06; $P = 0.23$). On the other hand, *ACE* expression in adipose tissue and skin was positively correlated (Spearman Rho = 0.27; $P = 2.21 \times 10^{-7}$), with no significant correlation across the remaining tissues (Supplementary Figure 4).

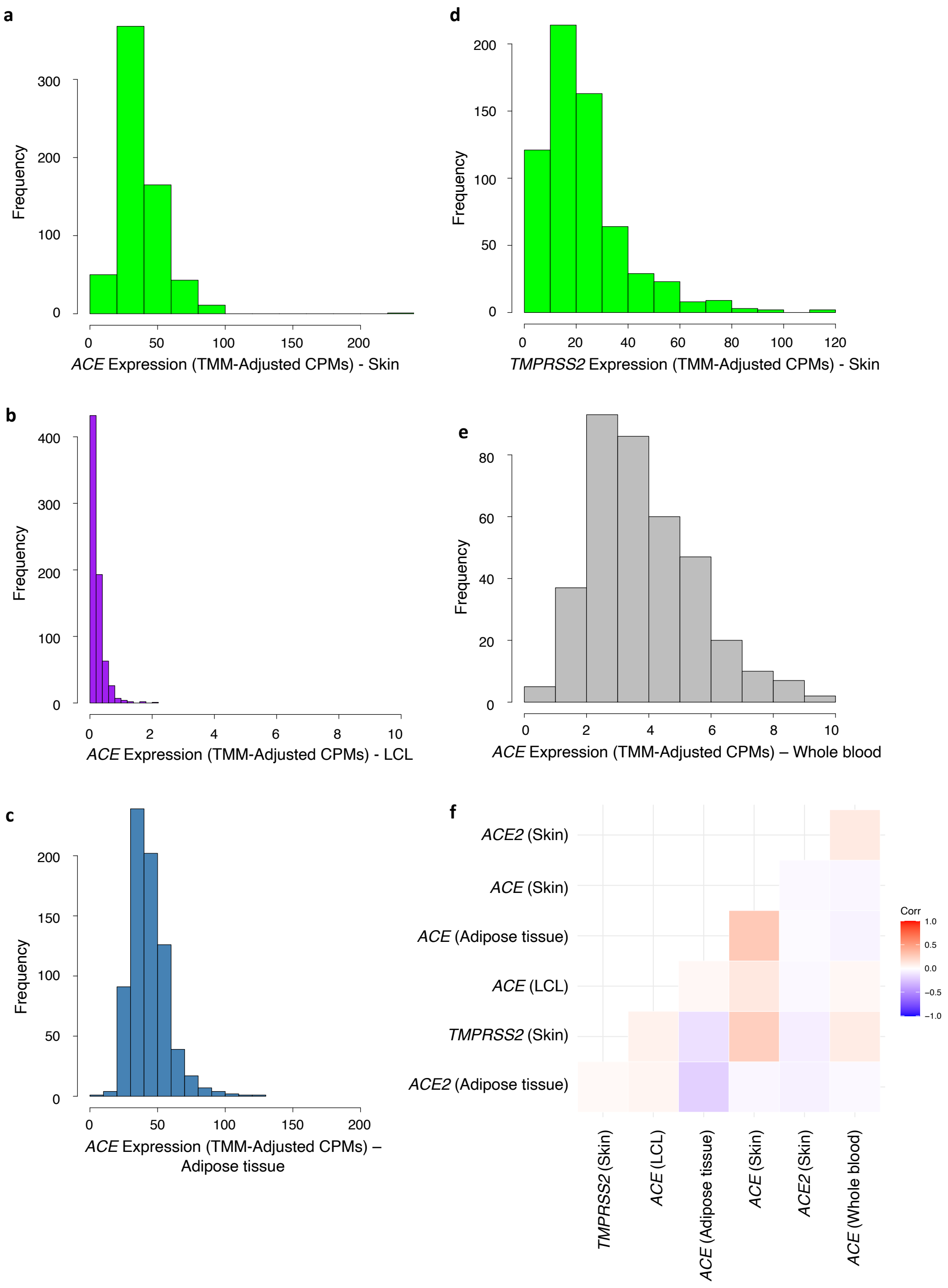

Supplementary Figure 4: Expression levels of RAS genes *ACE* and *TMPRSS2* across tissues in TwinsUK. a-e) Histograms of expression levels of ACE and TMPRSS2 expression in TwinsUK skin, LCL whole blood, and adipose tissue, as indicated. f) Correlation plot of RAAS gene expression across tissues in unrelated subjects from the TwinsUK study (n=463 participants).

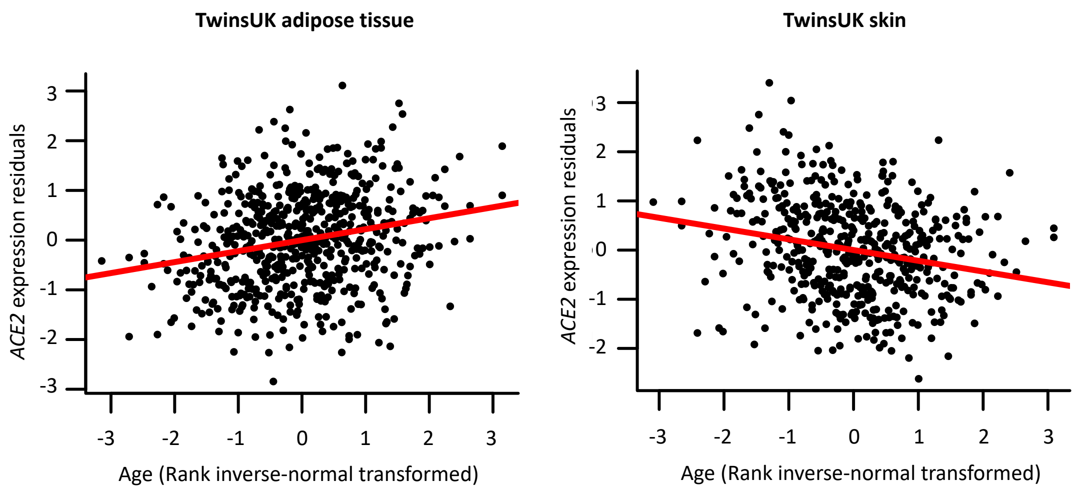

Supplementary Figure 5: *ACE2* expression in adipose tissue and skin show inverse direction of association with age. a) TwinsUK adipose tissue. b) TwinsUK skin. Each point represents an individual. Participant age was rank inverse-normal transformed. *ACE2* expression residuals plotted were adjusted for technical residuals and BMI to correspond to the reported association model.

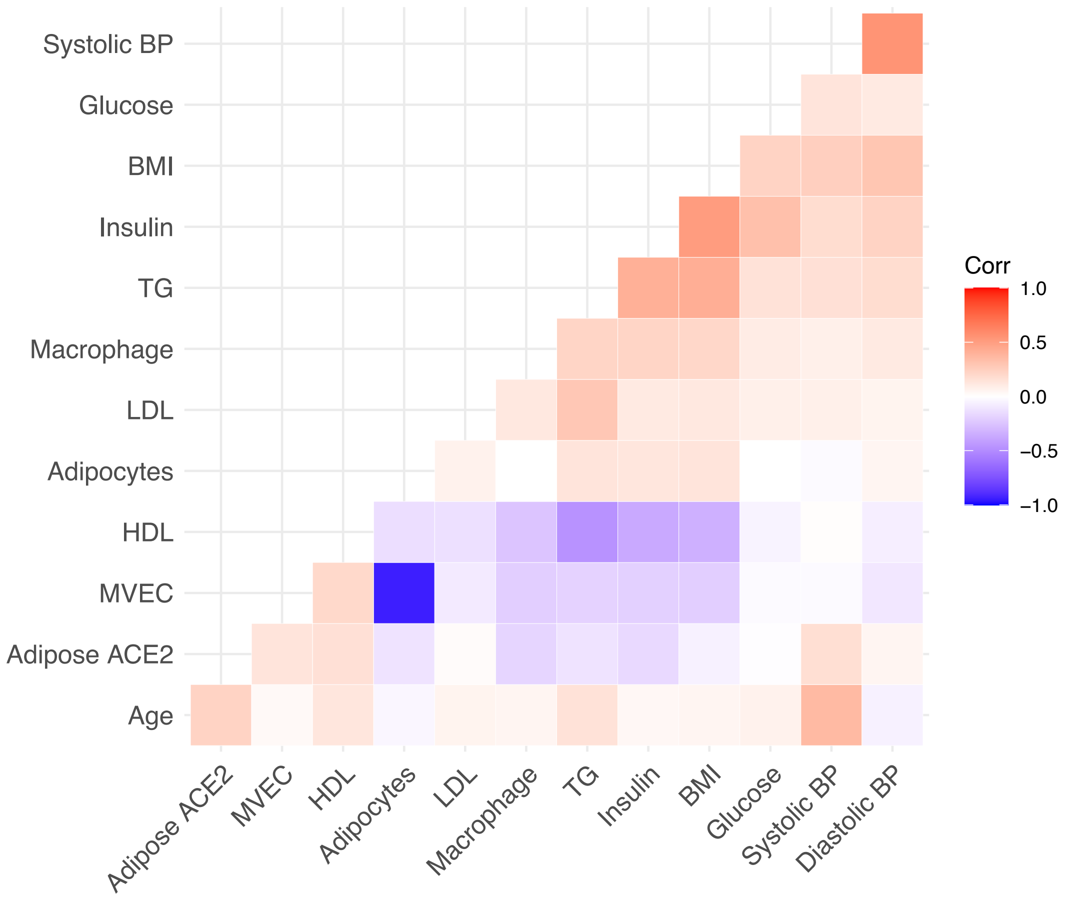

Supplementary Figure 6: Correlation plot of adipose tissue *ACE2* expression, phenotypic variables, and adipose tissue estimated cell type compositions in TwinsUK. Adipose tissue *ACE2* expression residuals were adjusted for RNASeq technical covariates**.** Correlations were assessed in unrelated subjects (n=441).

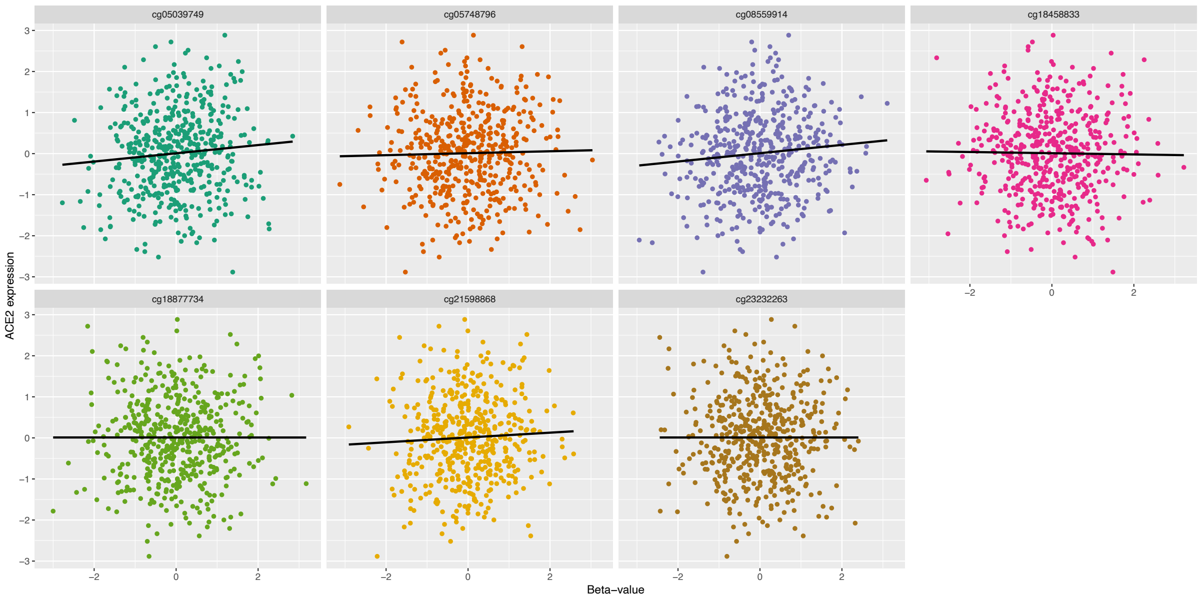

Supplementary Figure 7: Adipose tissue DNA methylation at methylation probes proximal to the *ACE2* gene is not associated with *ACE2* expression levels. Each point represents a single subject, with adipose tissue methylation beta values plotted on the x-axis and adipose tissue *ACE2* expression on the y-axis. Rank-based inverse normal transformation was applied to both methylation and gene expression data.

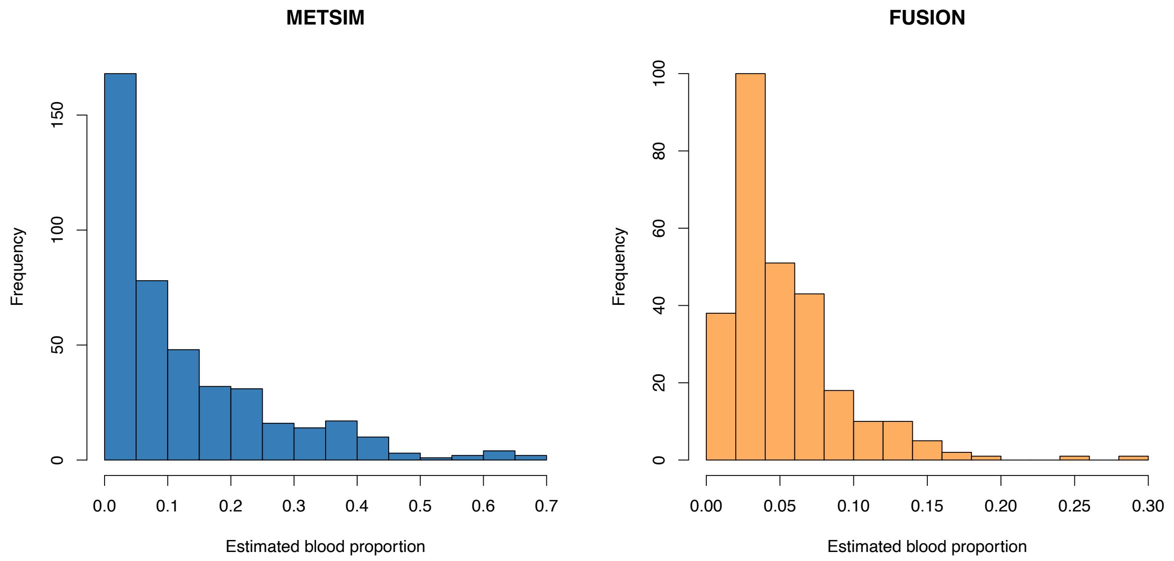

Supplementary Figure 8: Estimated cell type proportion of whole blood in the METSIM and FUSION studies. Cell type proportions were estimated using CIBERSORT (METSIM) and DESeq2 (FUSION) (See methods).
